# Peasecod‐Like Hollow Upconversion Nanocrystals with Excellent Optical Thermometric Performance

**DOI:** 10.1002/advs.202000731

**Published:** 2020-06-11

**Authors:** Huhui Fu, Caiping Liu, Pengfei Peng, Feilong Jiang, Yongsheng Liu, Maochun Hong

**Affiliations:** ^1^ State Key Laboratory of Structural Chemistry Fujian Institute of Research on the Structure of Matter Chinese Academy of Sciences Fuzhou Fujian 350002 China

**Keywords:** hollow nanocrystals, lanthanide ions, Li_4_ZrF_8_, nanothermometers, upconversion

## Abstract

Trivalent lanthanide (Ln^3+^)‐doped hollow upconversion nanocrystals (UCNCs) usually exhibit unique optical performance that cannot be realized in their solid counterparts, and thus have been receiving tremendous interest from their fundamentals to diverse applications. However, all currently available Ln^3+^‐doped UCNCs are solid in appearance, the preparation of hollow UCNCs remains nearly untouched hitherto. Herein, a class of UCNCs based on Yb^3+^/Er^3+^‐doped tetralithium zirconium octafluoride (Li_4_ZrF_8_:Yb/Er) featuring 2D layered crystal lattice is reported, which makes the fabrication of hollow UCNCs with a peasecod‐like shape possible after Ln^3+^ doping. By employing the first‐principle calculations, the unique peasecod‐like hollow nanoarchitecture primarily associated with the hetero‐valence Yb^3+^/Er^3+^ doping into the 2D layered crystal lattice of Li_4_ZrF_8_ matrix is revealed. Benefiting from this hollow nanoarchitecture, the resulting Li_4_ZrF_8_:Yb/Er UCNCs exhibit an abnormal green upconversion luminescence in terms of the population ratio between two thermally coupled states (^2^H_11/2_ and ^4^S_3/2_) of Er^3+^ relative to their solid Li_2_ZrF_6_:Yb/Er counterparts, thereby allowing to prepare the first family of hollow Ln^3+^‐doped UCNCs as supersensitive luminescent nanothermometer with almost the widest temperature sensing range (123–800 K). These findings described here unambiguously pave a new way to fabricate hollow Ln^3+^‐doped UCNCs for numerous applications.

## Graphical abstract

A class of peasecod‐like hollow Yb^3+^/Er^3+^‐doped Li_4_ZrF_8_ upconverting nanocrystals (UCNCs) that features a 2D layered crystal lattice is reported. Abnormal green upconverting luminescence that cannot be achieved in the solid UCNCs is observed in these peasecod‐like hollow UCNCs, thereby making them suitable as supersensitive nanothermometers with a wide temperature range for optical temperature sensing.

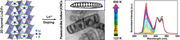

## 1 Introduction

**Figure 1:**
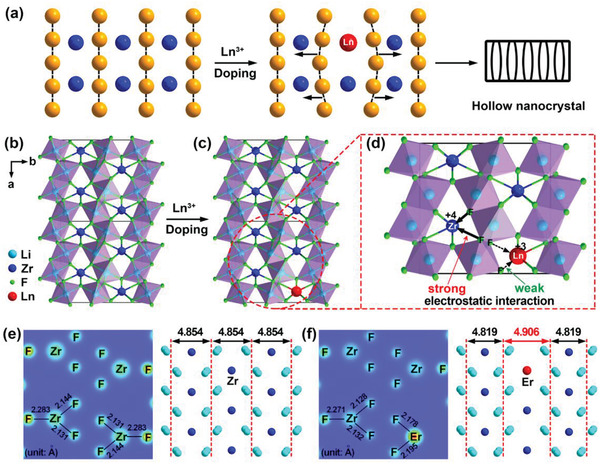
a) Proposed mechanism for the formation of hollow nanocrystals through Ln^3+^ doping in a 2D layered crystal structure. b) Crystal structure of orthorhombic‐phase Li_4_ZrF_8_ and c,d) the plausible localized crystal structure showing the different electrostatic interactions among Zr, Ln, and F atoms after the hetero‐valence Ln^3+^ doping in the lattice of Li_4_ZrF_8_ host matrix. The solid and dotted black arrows represent strong and weak electrostatic interactions, respectively. Comparison of electronic charge density for the e) pure and f) Er‐doped Li_4_ZrF_8_ crystals based on the first‐principle calculations, showing the slightly shortened Zr—F bonds in comparison with their pure counterparts after Ln^3+^ doping, and thus the interlayer dilation around the substituted Er^3+^ dopant.

Trivalent lanthanide (Ln^3+^) ions doped inorganic upconversion nanocrystals (UCNCs), possessing outstanding optical properties including large anti‐Stokes shifts, long excited‐state lifetimes and tunable emission colors, have drawn increasing attention for their potential applications in areas as diverse as biological imaging, detection, photonics, and temperature sensing.^[^ ^1^, ^2^, ^3^, ^4^, ^5^, ^6^, ^7^, ^8^, ^9^, ^10^, ^11^, ^12^, ^13^ ^]^ Particularly, with the rapid development of the nanocrystal synthesis technology, Ln^3+^‐doped UCNCs can now be prepared with finely controlled composition, phase, morphology, size, and output color.^[^ ^14^, ^15^, ^16^, ^17^, ^18^, ^19^, ^20^, ^21^, ^22^, ^23^ ^]^ However, as exemplified by the most representative *β*‐NaYF_4_:Yb/Er UCNCs (Figure S1, Supporting Information), all the currently available UCNCs are solid in appearance, which is primarily ascribed to their 3D close‐packed crystal structures that make the preparation of hollow Ln^3+^‐doped UCNCs impossible. In contrast, it is expected that the hollow nanostructures can be formed through hetero‐valence Ln^3+^ doping in a 2D layered crystal structure, where the interlayer dilation of crystal lattice induced by different electrostatic interactions can occur and thus trigger the formation of hollow Ln^3+^‐doped UCNCs (**Figure** **1**a), as achieved in other 2D porous nanomaterials such as carbon nanosheets,^[^ ^24^, ^25^ ^]^ graphitic carbon nitride nanosheets,^[^ ^26^, ^27^ ^]^ and metal chalcogenide nanocrystals.^[^ ^28^, ^29^ ^]^ Therefore, seeking an appropriate host material that features a 2D layered crystal structure is of key importance for the fabrication of hollow Ln^3+^‐doped UCNCs. It is believed that such hollow Ln^3+^‐doped UCNCs will exhibit a totally different optical performance that cannot be realized in their solid counterparts, owing to their superhigh surface‐to‐volume (*S*/*V*) ratio.

To this end, herein, we report the first class of hollow inorganic UCNCs based on Yb^3+^/Er^3+^‐doped tetralithium zirconium octafluoride (hereafter referred to as Li_4_ZrF_8_:Yb/Er) that is characterized by a 2D layered crystal structure (Figure 1b). This unique 2D layered crystal structure of inorganic Li_4_ZrF_8_ fluoride enables the preparation of hollow Ln^3+^‐doped UCNCs after the hetero‐valence doping of Yb^3+^/Er^3+^ into the Li_4_ZrF_8_ host matrix (Figure 1c,d). By employing the first‐principle calculations based on density functional theory (DFT), we provide direct and solid evidence to support that the hollow nanostructure of Li_4_ZrF_8_:Yb/Er UCNCs is primarily due to the interlayer dilation of crystal lattice, induced by the hetero‐valence doping of Yb^3+^/Er^3+^ into the 2D layered crystal lattice of Li_4_ZrF_8_ host. Thanks to the novel hollow nanostructure, an abnormal upconversion luminescence (UCL) property is observed in these Li_4_ZrF_8_:Yb/Er UCNCs when compared with their solid counterparts, thereby allowing us to fabricate the first family of hollow Ln^3+^‐doped UCNCs that can serve as promising luminescent nanothermometers with excellent optical thermometric performance.

## 2 Results and Discussion

In our design, orthorhombic‐phase Li_4_ZrF_8_ was chosen as the host material for the synthesis of hollow Ln^3+^‐doped UCNCs due to its unique 2D layered crystal structure. The Li_4_ZrF_8_ has an orthorhombic crystal structure (space group *Pnma*, *a* = 9.738 Å, *b* = 9.747 Å, *c* = 5.758 Å, *Z* = 4, ICSD No. 80 398) with Zr^4+^ ion separated by LiF_6_ octahedra that form the corrugated infinite layers, resulting in an overall 2D layered crystal structure. On this basis, we recognize that the hollow Li_4_ZrF_8_:Yb/Er UCNCs can be achieved after the hetero‐valence doping of Ln^3+^ into the crystal lattice of Li_4_ZrF_8_, owing to the different electrostatic interactions between the strong Zr—F and weak Ln‐F bonds, as clearly indicated in Figure 1c,d. This can be well substantiated by means of the first‐principle calculations based on DFT, where almost all the Zr—F bonds (2.271, 2.128, and 2.132 Å) in the Li_4_ZrF_8_:Yb/Er UCNCs were calculated to be slightly shorter than those (2.283, 2.144, and 2.131 Å) in their pure Li_4_ZrF_8_ counterparts (Figure 1e,f and Table S1, Supporting Information), and thereby leading to a broadened interlayer spacing of the LiF_6_ octahedra layers from 4.854 Å for the pure Li_4_ZrF_8_ to 4.906 Å for the Er‐doped Li_4_ZrF_8_ (Figure 1e,f and Figure S2, Supporting Information). To probe the feasibility of such hetero‐valence Yb^3+^/Er^3+^ doping into the lattice of Li_4_ZrF_8_ NCs, the crystal lattice parameters and formation energies of pure, Yb^3+^ and/or Er^3+^ doped Li_4_ZrF_8_ NCs were further determined, assumed that Yb^3+^ and Er^3+^ ions are located at the substitutional Zr^4+^ lattice sites in the orthorhombic Li_4_ZrF_8_ crystal with an overall doping content of 12.5 mol% (Tables S2 and S3, Supporting Information). Although no apparent change in the crystal lattice parameters was observed for pure and Yb^3+^/Er^3+^‐doped Li_4_ZrF_8_ NCs, the formation energy per atom was calculated to decrease by about 0.07 eV when Zr^4+^ ions were partially replaced by Yb^3+^/Er^3+^couple, clearly indicating that the substitution of Zr^4+^ by Yb^3+^/Er^3+^ in the lattice of Li_4_ZrF_8_ NCs is thermodynamically favored regardless of their discrepancy in the valence state and ionic radius (0.84 Å for Zr^4+^, 0.99 Å for Yb^3+^ and 1.00 for Er^3+^ with a coordination number of eight).^[^ ^30^ ^]^

**Figure 2:**
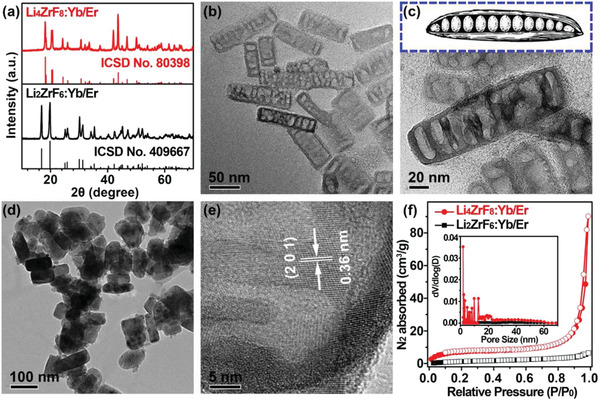
a) Powder XRD patterns of the as‐synthesized Li_4_ZrF_8_:Yb/Er and Li_2_ZrF_6_:Yb/Er UCNCs. b,c) TEM images of the hollow Li_4_ZrF_8_:Yb/Er UCNCs at different magnifications. The inset of c) schematically illustrates a peasecod‐like shape of the hollow Li_4_ZrF_8_:Yb/Er UCNCs. d) TEM image of the solid Li_2_ZrF_6_:Yb/Er UCNCs. e) High‐resolution TEM image of the hollow Li_4_ZrF_8_:Yb/Er UCNCs. f) N_2_ adsorption–desorption isotherms and corresponding pore‐size distributions (inset) for the hollow Li_4_ZrF_8_:Yb/Er (red) and solid Li_2_ZrF_6_:Yb/Er (black) UCNCs.

On the basis of these first‐principle calculations, we then doped the typical upconverting lanthanide couple of Yb^3+^/Er^3+^ at precisely defined doping concentration (20/2 mol%) into the lattice of monodisperse Li_4_ZrF_8_ NCs via a modified high temperature coprecipitation method as previously reported.^[^ ^31^ ^]^ For comparison, Yb^3+^/Er^3+^ couple was also introduced into the host matrix of 3D close‐packed dilithium hexafluorozirconate (Li_2_ZrF_6_) possessing identical elemental compositions but different stoichiometric ratios as compared with Li_4_ZrF_8_ by using similar synthetic procedures (Figure S3, Supporting Information). All the as‐synthesized Li_4_ZrF_8_:Yb/Er and Li_2_ZrF_6_:Yb/Er UCNCs can be indexed in accordance with orthorhombic‐phase Li_4_ZrF_8_ (ICSD No. 80 398) and monoclinic‐phase Li_2_ZrF_6_ (ICSD No. 409 667) crystals, respectively (**Figure** **2**a). Representative electron microscopy (TEM) images reveal that the as‐synthesized Li_4_ZrF_8_:Yb/Er UCNCs have a well‐defined peasecod‐like shape with an average length of 100 ± 15.2 nm and a width of 25 ± 5.1 nm (Figure 2b,c), and of which a large amount of internal cavities with an estimated pore size of 5–40 nm are clearly detected (Figure 2c), totally different from the case of Li_2_ZrF_6_:Yb/Er UCNCs possessing solid nanostructures (Figure 2d). Note that similar hollow nanostructures can be also formed in other Ln^3+^ ions (Yb/Tm, Yb/Ho, and Eu) doped Li_4_ZrF_8_ nanocrystals (Figure S4, Supporting Information). Regardless of their unique hollow nanoarchitecture formed, clear lattice fringes with a d‐spacing of 0.36 nm are observed at the edge of these internal cavities of peasecod‐like Li_4_ZrF_8_:Yb/Er UCNCs (Figure 2e), in good agreement with the lattice spacing of (201) plane of orthorhombic‐phase Li_4_ZrF_8_ crystal (ICSD No. 80 398) and thus reveals the formation of highly crystalline Li_4_ZrF_8_:Yb/Er UCNCs. Compositional analyses by energy‐dispersive X‐ray spectroscopy coupled with inductively coupled plasma atomic emission spectroscopy confirm the presence of host elements of Zr and F and the dopants of Yb and Er (Figure S5 and Table S4, Supporting Information), which clearly demonstrates the successful doping of Yb^3+^ and Er^3+^ ions in the as‐synthesized hollow Li_4_ZrF_8_:Yb/Er and solid Li_2_ZrF_6_:Yb/Er UCNCs.

To shed more light on the hollow Li_4_ZrF_8_:Yb/Er UCNCs, we further carried out the N_2_ adsorption‐desorption experiments on Li_4_ZrF_8_:Yb/Er and Li_2_ZrF_6_:Yb/Er UCNCs. As compared in Figure 2f, the Brunauer–Emmet–Teller (BET) surface area and pore volume for the hollow Li_4_ZrF_8_:Yb/Er UCNCs were calculated to be 46.2 m^2^ g^−1^ and 0.07 cm^3^ g^−1^, which are about eleven and nine times larger than those of their solid Li_2_ZrF_6_:Yb/Er counterparts (4.1 m^2^ g^−1^ and 0.008 cm^3^ g^−1^), respectively. As a result, pores with sizes in the range of 2–25 nm can be clearly detected in the pore‐size distribution of Li_4_ZrF_8_:Yb/Er UCNCs, which differs markedly from the case of solid Li_2_ZrF_6_:Yb/Er UCNCs showing no any pores (Inset of Figure 2f). Note that the hollow nanostructure cannot come into being in the pure Li_4_ZrF_8_ NCs under otherwise identical synthetic conditions (Figure S6, Supporting Information), such significantly increased BET surface area and pore volume for the Li_4_ZrF_8_:Yb/Er UCNCs relative to the Li_2_ZrF_6_:Yb/Er counterparts unambiguously demonstrate that the hollow nanostructure observed in the Li_4_ZrF_8_:Yb/Er UCNCs is indeed originating from the Ln^3+^‐doping in the 2D layered crystal structure of Li_4_ZrF_8_, consistent well with our theoretical first‐principle calculations based on DFT (Figure 1e,f).

**Figure 3:**
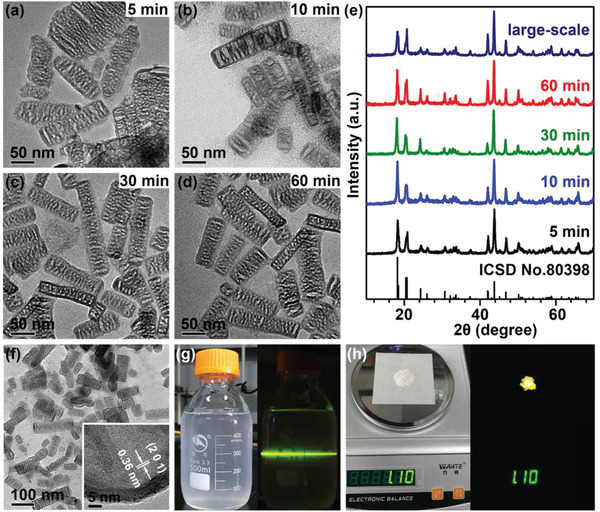
a–d) TEM images of the hollow Li_4_ZrF_8_:Yb/Er UCNCs prepared in different reaction time of 5, 10, 30, and 60 min, respectively. e) XRD patterns of the hollow Li_4_ZrF_8_:Yb/Er UCNCs prepared in different reaction time and via a large‐scale synthesis. f) TEM and high‐resolution TEM (inset) images of the hollow Li_4_ZrF_8_:Yb/Er UCNCs via the large‐scale synthesis. g) Photographs of the hollow Li_4_ZrF_8_:Yb/Er UCNCs via the large‐scale synthesis dispersed in 500 mL cyclohexane under daylight (left) and upon excitation at 980 nm (right). h) Photographs showing the weight (1.1 g) of the resulting hollow UCNCs via a one‐pot synthesis under daylight (left) and UCL upon irradiation at 980 nm (right), respectively.

Another prominent feature for these hollow Li_4_ZrF_8_:Yb/Er UCNCs is that they can be rapidly synthesized in a relatively short period of time of ≈5 min. **Figure** **3**a–d show the typical TEM images of the hollow Li_4_ZrF_8_:Yb/Er UCNCs prepared in various reaction time of 5, 10, 30, and 60 min, respectively. Despite the dramatically shortened reaction time, the well‐defined hollow nanostructure for the peasecod‐like Li_4_ZrF_8_:Yb/Er UCNCs prepared in 5 min keeps essentially identical to those synthesized at prolonged reaction time ranging from 10 to 60 min. Particularly, in all cases, their XRD peaks for Li_4_ZrF_8_:Yb/Er UCNCs prepared in different reaction time match the standard orthorhombic‐phase Li_4_ZrF_8_ crystal in terms of line position and full width at half maximum, which strongly support that the formation of hollow Li_4_ZrF_8_:Yb/Er UCNCs can be quickly completed in a very short time down to ≈5 min (Figure 3e). In addition to the rapid synthesis, we would like to emphasize that the hollow Li_4_ZrF_8_:Yb/Er UCNCs can be also prepared in large scale by only scaling up the reaction variables such as the amount of Zr precursor. In this way, over 1.1 g of Li_4_ZrF_8_:Yb/Er UCNCs were synthesized via a one‐pot reaction, where the crystal phase, morphology, nanocrystal size as well as the UCL performance for the resulting Li_4_ZrF_8_:Yb/Er UCNCs were found to be preserved during scaling up (Figure 3e–h and Figure S7, Supporting Information).

**Figure 4:**
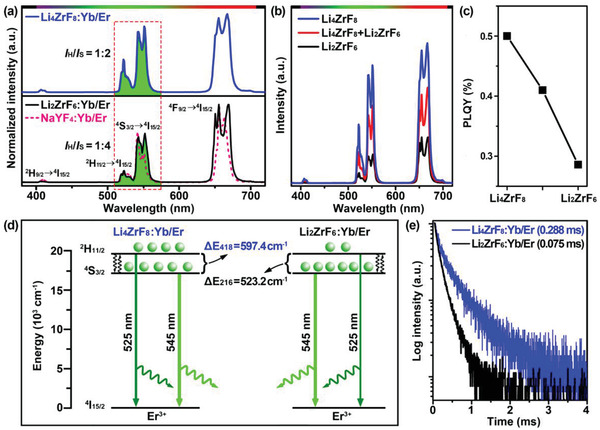
a) Comparison of typical UCL spectra for the hollow Li_4_ZrF_8_:Yb/Er, solid Li_2_ZrF_6_:Yb/Er, and solid *β*‐NaYF_4_:Yb/Er UCNCs under excitation of a 980‐nm diode laser. b) Comparison of UCL spectra for the hollow Li_4_ZrF_8_:Yb/Er, solid Li_2_ZrF_6_:Yb/Er, and the mixed‐phase UCNCs, and c) their corresponding PLQYs, demonstrating the gradually increased UCL performance from the solid Li_2_ZrF_6_:Yb/Er to hollow Li_4_ZrF_8_:Yb/Er UCNCs. d) Proposed mechanism for green UC emissions assigned to the ^2^H_11/2_→^4^I_15/2_ (centered at 525 nm) and ^4^S_3/2_→^4^I_15/2_ (centered at 545 nm) transitions of Er^3+^, showing their different population ratios for the ^2^H_11/2_ and ^4^S_3/2_ states of Er^3+^ in the hollow Li_4_ZrF_8_:Yb/Er and solid Li_2_ZrF_6_:Yb/Er UCNCs, respectively. e) Comparison of the ^2^H_11/2_ lifetime of Er^3+^ in the hollow Li_4_ZrF_8_:Yb/Er and solid Li_2_ZrF_6_:Yb/Er UCNCs, respectively.

**Figure** **4**a compares the representative room‐temperature UCL spectra for the hollow Li_4_ZrF_8_:Yb/Er, solid Li_2_ZrF_6_:Yb/Er and solid *β*‐NaYF_4_:Yb/Er UCNCs when excited by using a 980‐nm diode laser at a power density of 10 W cm^−2^. The characteristic UCL bands arising from the ^2^H_9/2_→^4^I_15/2_ (406 nm), ^2^H_11/2_→^4^I_15/2_ (525 nm), ^4^S_3/2_→^4^I_15/2_ (545 nm), and ^4^F_9/2_→^4^I_15/2_ (650 nm) transitions of Er^3+^ are readily detected in the visible spectral regions for both UCNCs. Regardless of their much larger *S*/*V* ratio, the overall UCL intensity for hollow Li_4_ZrF_8_:Yb/Er UCNCs was determined to be about 3.4 times stronger than that of solid Li_2_ZrF_6_:Yb/Er samples (Figure 4b and Figure S8, Supporting Information), which differs markedly from the previous observation showing that the large *S*/*V* ratio of Ln^3+^‐doped UCNCs usually leads to weak UCL due to severe luminescence quenching.^[^ ^32^ ^]^ Accordingly, the photoluminscence quantum yield (PLQY) was observed to stepwise decrease with the phase transformation from hollow Li_4_ZrF_8_:Yb/Er (≈0.50%) to solid Li_2_ZrF_6_:Yb/Er (≈0.29%) UCNCs (Figure 4c and Figure S8, Supporting Information). Especially, the integrated luminescence intensity ratio (LIR) between these two thermally coupled levels (^2^H_11/2_ and ^4^S_3/2_) of Er^3+^ in the hollow Li_4_ZrF_8_:Yb/Er UCNCs (termed as *I*_H_/*I*_S_) was determined to 1/2, which is about two times larger than that observed in the solid Li_2_ZrF_6_:Yb/Er and *β*‐NaYF_4_:Yb/Er controls (1/4, Figure 4a). This experimental observation strongly supports that the population ratio for the ^2^H_11/2_ state to ^4^S_3/2_ state of Er^3+^ in the hollow Li_4_ZrF_8_:Yb/Er UCNCs is much larger than in the solid Li_2_ZrF_6_:Yb/Er ones (Figure 4d), as further evidenced by the significantly prolonged ^2^H_11/2_ lifetime of Er^3+^ from the solid Li_2_ZrF_6_:Yb/Er (≈0.08 ms) to hollow Li_4_ZrF_8_:Yb/Er (≈0.29 ms) UCNCs (Figure 4e).

Owing to the small energy gap (Δ*E*) between the two energy levels, in theory, the ^2^H_11/2_ level of Er^3+^ can be easily populated from its low‐lying ^4^S_3/2_ state via thermal excitation. Consequently, the population ratio for the ^2^H_11/2_ state to ^4^S_3/2_ state of Er^3+^ can be described by the Boltzmann's distribution (∝‐Δ*E*/*K*_B_ *T*, here *K*_B_ is the Boltzmann's constant and *T* is the temperature in Kelvins) and thus scale linearly with the integrated LIR of *I*_H_/*I*_S_.^[^ ^33^, ^34^, ^35^, ^36^ ^]^ For Er^3+^ in the hollow Li_4_ZrF_8_:Yb/Er and solid Li_2_ZrF_6_:Yb/Er UCNCs, the Δ*E*_s_ were calculated to be about 597.4 and 523.2 cm^−1^ from their respective UCL spectra (Figure 4a). Theoretically, the population ratio for the ^2^H_11/2_ state to ^4^S_3/2_ state of Er^3+^ in the hollow Li_4_ZrF_8_:Yb/Er UCNCs with enlarged Δ*E* should be smaller than that in their solid Li_2_ZrF_6_:Yb/Er counterparts, which is obviously contrary to our experimental finding abovementioned (Figure 4d). Considering their completely identical elemental compositions of Li, Zr, and F for the hollow Li_4_ZrF_8_:Yb/Er and solid Li_2_ZrF_6_:Yb/Er UCNCs, we attribute this unusual increase in the LIR of *I*_H_/*I*_S_ to the hollow nanostructure of the peasecod‐like Li_4_ZrF_8_:Yb/Er UCNCs. To reconfirm this effect of the hollow nanostructure, we further synthesized the solid *β*‐NaYF_4_:Yb/Er (20/2 mol%) UCNCs as controls (Supporting Information). Indeed, the integrated LIR of *I*_H_/*I*_S_ and ^2^H_11/2_ lifetime for Er^3+^ in the solid *β*‐NaYF_4_:Yb/Er UCNCs were also found to be virtually identical to those of the solid Li_2_ZrF_6_:Yb/Er UCNCs (Figure 4a and Figure S9, Supporting Information), thereby providing another evidence to turn out that the increased LIR of *I*_H_/*I*_S_ is due to the hollow nanostructure formed in the peasecod‐like Li_4_ZrF_8_:Yb/Er UCNCs.

**Figure 5:**
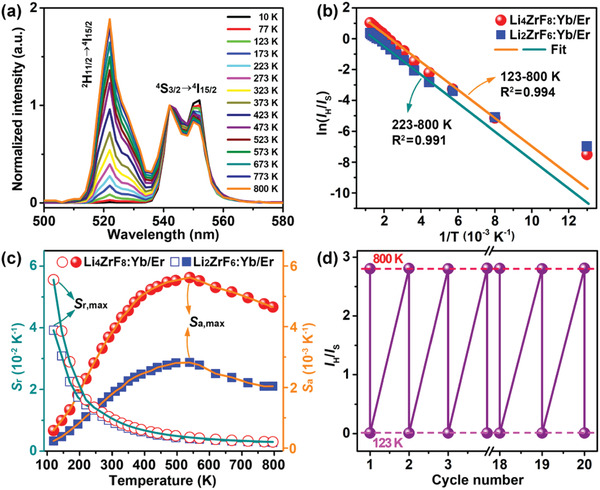
a) Temperature‐dependent UCL spectra for the hollow Li_4_ZrF_8_:Yb/Er UCNCs recorded at the temperature range of 10–800 K. The peaks are normalized at 542 nm. b) Plots of ln(*I*_H_/*I*_S_) versus 1/*T* and the fitted curves to calibrate the thermometric scale for the hollow Li_4_ZrF_8_:Yb/Er and solid Li_2_ZrF_6_:Yb/Er UCNCs, respectively. c) Calculated temperature sensitivities (*S*_a_ and *S*_r_) versus *T* in 123–800 K for the hollow Li_4_ZrF_8_:Yb/Er and solid Li_2_ZrF_6_:Yb/Er UCNCs, respectively. d) Variation of the intensity ratio *I*_H_/*I*_S_ measured at 123–800 K over a span of 20 cycles of heating and cooling processes for the hollow Li_4_ZrF_8_:Yb/Er UCNCs.

In view of the unusual population ratio of the ^2^H_11/2_ state to ^4^S_3/2_ state of Er^3+^ associated with the hollow nanostructure, we reasoned that Er^3+^ ions in these peasecod‐like Li_4_ZrF_8_:Yb/Er UCNCs would be more sensitive to the temperature when compared with their solid counterparts. To verify the temperature effect on UCL, we recorded the temperature‐dependent UCL spectra for the hollow Li_4_ZrF_8_:Yb/Er and solid Li_2_ZrF_6_:Yb/Er UCNCs in the temperature range from 10 to 800 K. The intensity ratios of *I*_H_/*I*_S_ of Er^3+^ for both the Li_4_ZrF_8_:Yb/Er and Li_2_ZrF_6_:Yb/Er UCNCs increased with rising temperature, as a result of enhanced thermal population of the ^2^H_11/2_ state from the ^4^S_3/2_ state at higher temperature (**Figure** **5**a and Figure S10, Supporting Information). However, as compared in Figure 5b, the hollow Li_4_ZrF_8_:Yb/Er UCNCs exhibited an excellent linear relationship between the ln(*I*_H_/*I*_S_) and inverse temperature (1/*T*) in the temperature range from 123 to 800 K, much wider than that of their solid Li_2_ZrF_6_:Yb/Er counterparts (223–800 K). Note that such a linear range of 123–800 K for the hollow Li_4_ZrF_8_:Yb/Er UCNCs is among the widest temperature ranges for the Yb^3+^/Er^3+^ codoped luminescent nanothermometers previously reported (**Table** **1**).^[^ ^12^, ^37^, ^38^, ^39^, ^40^, ^41^, ^42^, ^43^ ^]^ In addition to the linear temperature response range, the thermal sensitivities for the hollow Li_4_ZrF_8_:Yb/Er UCNCs also outperform their solid Li_2_ZrF_6_:Yb/Er counterparts (Figure 5c). For example, the highest absolute temperature sensitivity (*S*_a_, defined as ∂LIR/∂T)^[^ ^44^, ^45^ ^]^ and highest relative temperature sensitivity (*S*_r_, defined as (1/LIR)(∂LIR/∂T))^[^ ^44^, ^45^ ^]^ for the hollow Li_4_ZrF_8_:Yb/Er UCNCs were calculated to be 0.52% K^−1^ at 523 K and 5.65% K^−1^ at 123 K, which are about two and 1.43 times higher than those of solid Li_2_ZrF_6_:Yb/Er UCNCs (0.26% K^−1^ at 523 K and 3.95% K^−1^ at 123 K). Particularly, when compared with other Yb^3+^/Er^3+^ codoped UCNCs, the thermal sensitivities of the hollow Li_4_ZrF_8_:Yb/Er UCNCs are also superior to the most previously reported temperature sensing systems (Table 1).^[^ ^12^, ^37^, ^38^, ^39^, ^40^, ^41^, ^42^, ^43^ ^]^

**Table 1:** Comparison of various Yb^3+^/Er^3+^ co‐doped UCNCs as luminescent nanothermometers by using LIR technique in the green spectral region

Material	Temperature range [K]	*S*_a_, _max_ [% K^−1^]	*S*_r, max_ [% K^−1^]	References
Li_4_ZrF_8_:Yb/Er	123–800	0.52	5.65	This work
Li_2_ZrF_6_:Yb/Er	123–800	0.26	3.95	This work
KMnF_3_:Yb/Er	303–343	1.13	5.7	[37]
NaYF_4_:Yb/Er	173–353	–	3.58	[38]
CaF_2_:Yb/Er	293–318	–	2.3	[39]
Gd_2_O_3_:Yb/Er	300–900	0.39	0.83	[40]
SrF_2_:Yb/Er	303–373	–	1.21	[12]
Ba_5_Gd_8_Zn_4_O_21_:Yb/Er	260–490	0.32	1.69	[41]
csUCNP@C	293–343	–	1.1	[42]
YF_3_:Yb/Er	260–490	0.26	1.48	[43]

Moreover, such temperature evolution of *I*_H_/*I*_S_ for the hollow Li_4_ZrF_8_:Yb/Er UCNCs was found to be reversible during the heating and cooling cycles in the temperature range of 123–800 K. The intensity ratios of *I*_H_/*I*_S_ recorded at 123 and 800 K are nearly unchanged with deviations smaller than 0.5% over a span of 20 cycles during heating and cooling processes, indicative of the high reliability of these hollow Li_4_ZrF_8_:Yb/Er UCNCs as optical thermometric materials (Figure 5d). Taken together, these results unambiguously demonstrate that the as‐synthesized peasecod‐like hollow Li_4_ZrF_8_:Yb/Er UCNCs are highly promising candidates as luminescent nanothermometers for optical temperature sensing. Currently, further efforts on the practical use of these hollow nanocrystals as in vivo temperature sensor or drug delivery carrier are underway in our laboratory.

## 3 Conclusion

In summary, we reported for the first time a peasecod‐like hollow Li_4_ZrF_8_:Yb/Er UCNCs that were synthesized through a modified high‐temperature coprecipitation method. By utilizing the first‐principle calculations based on DFT, we revealed that the hollow nanostructure of Li_4_ZrF_8_:Yb/Er UCNCs arouse primarily from the hetero‐valence substituted doping of Ln^3+^ into the Li_4_ZrF_8_ host lattice that features a 2D layered crystal structure. Furthermore, these peasecod‐like hollow Li_4_ZrF_8_:Yb/Er UCNCs were able to be prepared rapidly and in large scale with preserved crystal phase, morphology, size, and UCL properties. Benefiting from the hollow nanostructure, these Li_4_ZrF_8_:Yb/Er UCNCs exhibited an abnormal UCL performance in comparison with their solid counterparts, thereby endowing them with excellent optical thermometric performances including a wide temperature range, relatively high thermal sensitivity and high photochemical stability. These findings unambiguously pave a new way to construct the hollow Ln^3+^‐doped inorganic UCNCs for various applications such as optical temperature sensing and bioimaging.

## 4 Experimental Section

##### General Procedure for the Preparation of Peasecod‐Like Hollow Li_4_ZrF_8_:Yb/Er UCNCs

In a typical procedure for the synthesis of Li_4_ZrF_8_:Yb/Er UCNCs (1 mmol), 2 mmol of LiOH·2H_2_O, 0.78 mmol of Zr(CH_3_COO)_4_, 0.2 mmol of Yb(CH_3_COO)_3_·4H_2_O, and 0.02 mmol of Er(CH_3_COO)_3_·4H_2_O were mixed with 8 mL of OA and 16 mL of ODE in a 100 mL three‐neck round‐bottom flask. The solution was heated to 150 °C under N_2_ flow with constant stirring for 60 min to form a clear solution, and then cooled down to room temperature. Thereafter, 10 mL of methanol solution containing 3 mmol of NH_4_F was added and the resulting mixture was stirred for 30 min. After removal of the methanol by evaporation, the solution was heated to 280 °C under N_2_ flow with vigorous stirring for 60 min, and then cooled down to room temperature. The resulting Li_4_ZrF_8_:Yb/Er UCNCs were precipitated by addition of ethanol, collected by centrifugation, washed with ethanol and cyclohexane for several times, and finally redispersed in cyclohexane. As for the large‐scale synthesis of 20 mmol hollow Li_4_ZrF_8_:Yb/Er UCNCs, identical experimental procedures were used except for proportionately scaling up the reaction variables including the amount of precursors and solvents. In addition, the general procedures for the preparation of solid Li_2_ZrF_6_:Yb/Er and *β*‐NaYF_4_:Yb/Er UCNCs are provided in the Supporting Information.

##### Structural and Optical Characterization

Powder XRD measurements were performed on a powder diffractometer (MiniFlex2, Rigaku) with Cu K*α*1 radiation (*λ* = 0.154187 nm) from 10° to 70° at a scanning rate of 5° min^−1^. Both the TEM and high‐resolution TEM measurements were conducted on a TEM (TECNAI G2F20) equipped with an energy dispersive X‐ray spectroscope. The N_2_ adsorption‐desorption isotherms and BET surface area of samples were determined by gas sorption analysis (Micromeritics ASAP 2020, 77 K). UCL spectra were measured upon 980‐nm NIR excitation from a continuous‐wave diode laser. UCL photographs of the UCNCs were taken by using a Canon 70D digital camera without using any filter. All the UCL decay curves for Yb^3+^/Er^3+^ co‐doped UCNCs were measured with a customized UV to mid‐infrared steady‐state and phosphorescence lifetime spectrometer (FSP920‐C, Edinburgh Instrument) equipped with a digital oscilloscope (TDS3052B, Tektronix) and a tunable mid‐band Optical Parametric Oscillator (OPO) pulse laser as the excitation source (410–2400 nm, 10 Hz, pulse width ≤5 ns, Vibrant 355II, OPOTEK). For temperature‐dependent UCL measurements, the as‐synthesized UCNCs were put inside the Linkam THMS600E heating and freezing stage with a tunable temperature range from 10 to 800 K and heating rate of 60 °C min^−1^. The as‐prepared UCNCs were in situ cooled or heated on the stage, whose temperature was controlled to a certain temperature by a temperature controller (Linkam LNP95) and held for 5 min to keep the temperature stable, and then proceeded to a next temperature. All the spectral data were corrected for the spectral response of the spectrometer.

##### Computational Details

A theoretical assessment for the pure, Yb^3+^ and/or Er^3+^ doped Li_4_ZrF_8_ system was performed using the Vienna Ab initio Simulation Package (VASP).^[^ ^46^ ^]^ The generalized gradient approximation (GGA) with Perdew–Burke–Ernzerhof^[^ ^47^ ^]^ functional and the projector‐augmented‐wave pseudopotentials method were introduced to compute the exchange‐correlation and valence‐core interactions, respectively. The geometric optimizations and the formation energy (*E*_form_) of the pure and Yb^3+^ and/or Er^3+^ doped Li_4_ZrF_8_ NCs were first evaluated to confirm the successful hetero‐valence doping of Ln^3+^ ions in the Li_4_ZrF_8_ host lattice. To study the doping effect, the 1 × 1 × 2 supercell containing 104 atoms was constructed, in which one and two zirconium ions were replaced by Ln^3+^ ions with doping concentrations of 12.5% and 25% respectively. Considering the strongly‐correlated character of 4f valence electrons of Yb^3+^ and Er^3+^ ions, the Heyd–Scuseria–Ernzerhof hybrid functional (HSE06) or GGA+Umethods were adopted to provide more accurate electronic properties for Ln^3+^‐doped materials.^[^ ^48^, ^49^, ^50^ ^]^ Due to the large number of atoms, calculations with the HSE06 functional were crudely terminated. Therefore, all of the calculated results were obtained by using the GGA+*U* method. It should be mentioned that the *U*_eff_ values introduced by Dudarev were employed as the *U* values for Zr and Yb/Er atoms, in which three equations of *U*_eff_ = *U* − *J*, *J* = 0 and *U*_eff_ = *U* were set up and values of 2.0 and 6.0 eV were appointed to zirconium and lanthanide ions, respectively.^[^ ^51^ ^]^ The kinetic energy cutoff was set to 400 eV. All of the atomic positions and cell parameters were allowed to relax until the limits of 1 × 10^−6 ^eV for energy and −0.01 eV Å^−1^ for force, respectively. For the Brillouin zone sampling, the 3 × 3 × 3 Γ‐centered k‐point mesh was used for all calculations. Moreover, the post‐processing and graphics of the calculation results were completed by using the VASPKIT and VESTA softwares.^[^ ^52^, ^53^ ^]^

## Conflict of Interest

The authors declare no conflict of interest.

## Supporting information

Supporting Information
