# Supplementary material for: Peasecod‐Like Hollow Upconversion Nanocrystals with Excellent Optical Thermometric Performance
Source: Adv Sci (Weinh). 2020 Jun 11;7(14):2000731. doi: 10.1002/advs.202000731 (PMC7375223; doi:10.1002/advs.202000731)
Supplement: Supplementary file 1 — Supporting Information [file ADVS-7-2000731-s001.pdf]

Copyright WILEY-VCH Verlag GmbH & Co. KGaA, 69469 Weinheim, Germany, 2020.

Supporting Information

Peasecod-like Hollow Upconversion Nanocrystals with Excellent Optical Thermometric Performance

Huhui Fu, Caiping Liu, Pengfei Peng, Feilong Jiang, Yongsheng Liu, and Maochun Hong**

Experimental Section

Chemicals and Materials: LiOH, NaOH and NH_4F were purchased from Aladdin (China). $Y(CH_3CO_2)_3·4H_2O$ (99.999%), $Yb(CH_3CO_2)_3·4H_2O$ (99.999%), $Er(CH_3CO_2)_3·4H_2O$ (99.99%), Zirconium acetate solution, Oleic acid (OA) and 1-octadecene (ODE) were purchased from Sigma-Aldrich (China). Cyclohexane, methanol, and ethanol were purchased from Sinopharm Chemical Reagent Co., China. All chemicals were used as received without further purification.

General procedure for the preparation of solid Li_2ZrF_6:Yb/Er UCNCs: In brief, 2 mmol of $LiOH·2H_2O$, 0.78 mmol of $Zr(CH_3COO)_4$, 0.2 mmol of $Yb(CH_3COO)_3·4H_2O$ and 0.02 mmol of $Er(CH_3COO)_3·4H_2O$ were mixed with 8 mL of OA and 12 mL of ODE in a 100 mL three-neck round-bottom flask. The solution was heated to 150 °C under N_2 flow with constant stirring for 60 min to form a clear solution, and then cooled down to room temperature. Thereafter, 10 mL of methanol solution containing 6 mmol of NH_4F was added and the resulting mixture was stirred for 30 min. After removal of the methanol by evaporation, the solution was heated to 280 °C under N_2 flow with vigorous stirring for 60 min, and then cooled down to room temperature. The obtained Li_2ZrF_6:Yb/Er UCNCs were precipitated by addition of ethanol, collected by centrifugation, then washed with ethanol for several times, and finally redispersed in cyclohexane.

General procedure for the preparation of solid β-$NaYF_4$:Yb/Er UCNCs: In brief, 0.78 mmol of $Y(CH_3CO_2)_3 \cdot 4H_2O$, 0.2 mmol of $Yb(CH_3CO_2)_3 \cdot 4H_2O$ and 0.02 mmol of $Er(CH_3CO_2)_3 \cdot 4H_2O$ were mixed with 5 mL of OA and 16 mL of ODE in a 100 mL three-neck round-bottom flask. The solution was heated to 150 °C under N_2 flow with constant stirring for 60 min to form a clear solution, and then cooled down to room temperature. Thereafter, 10 mL of methanol solution containing 2.5 mmol of NaOH and 4 mmol of NH_4F was added and the resulting mixture was stirred for 30 min. After removal of the methanol by evaporation, the solution was heated to 300 °C under N_2 flow with vigorous stirring for 60 min, and then cooled down to room temperature. The obtained β-$NaYF_4$:Yb/Er UCNCs were precipitated by addition of ethanol, collected by centrifugation, washed with ethanol for several times, and finally redispersed in cyclohexane.

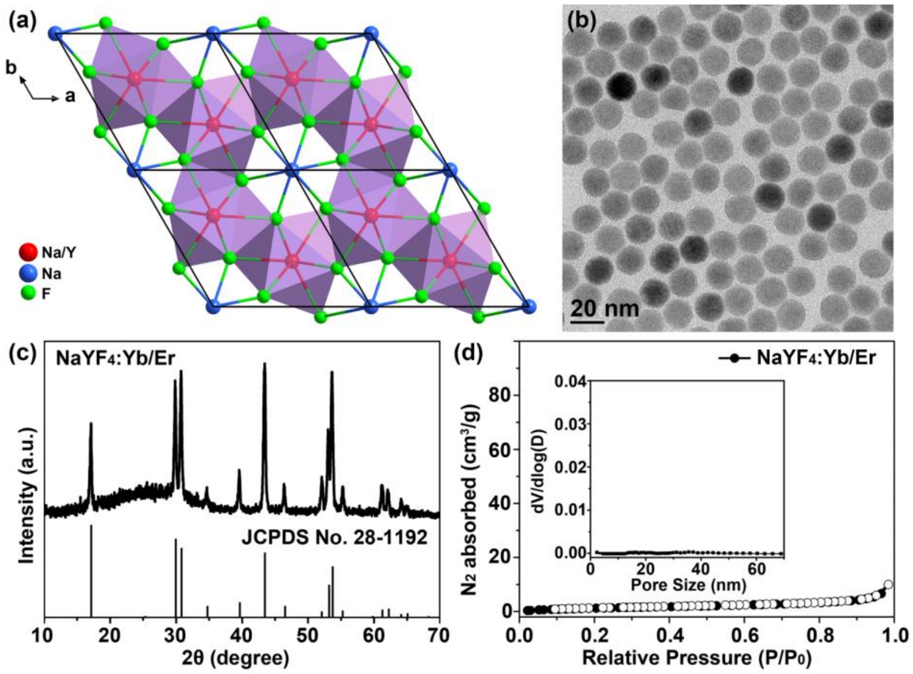

Figure S1. (a) Hexagonal-phase β-$NaYF_4$ that features a three-dimensional (3D) close-packed crystal structure. (b) TEM image of the as-prepared β-$NaYF_4$:Yb/Er UCNCs, appearing as solid nanospheres with an average size about 17 nm. (c) XRD pattern of β-$NaYF_4$:Yb/Er UCNCs, showing that all the diffraction peaks can be well indexed into hexagonal-phase β-$NaYF_4$ (JCPDS No. 28-1192). (d) N_2 adsorption-desorption isotherms and corresponding pore size distribution of β-$NaYF_4$:Yb/Er UCNCs, further demonstrating their solid nanostructures without any pores.

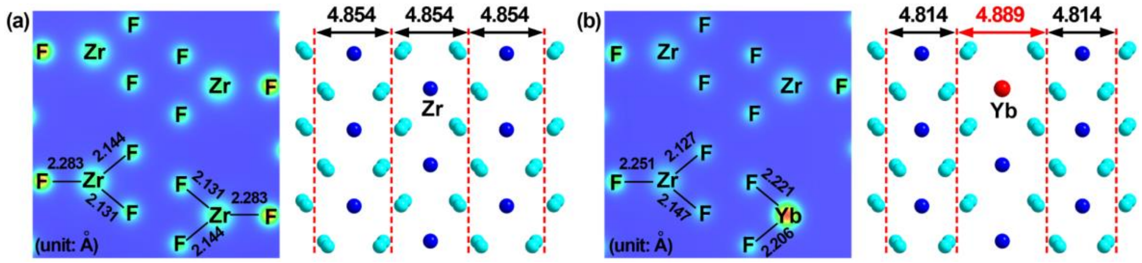

Figure S2. Comparison of electronic charge density for the (a) pure and (b) Yb-doped Li_4ZrF_8 crystals based on the first-principle calculations, showing the slightly shortened Zr-F bonds in comparison with their pure counterparts after Yb^{3+} doping, and thus leading to the interlayer dilation around the substituted Yb^{3+} dopant.

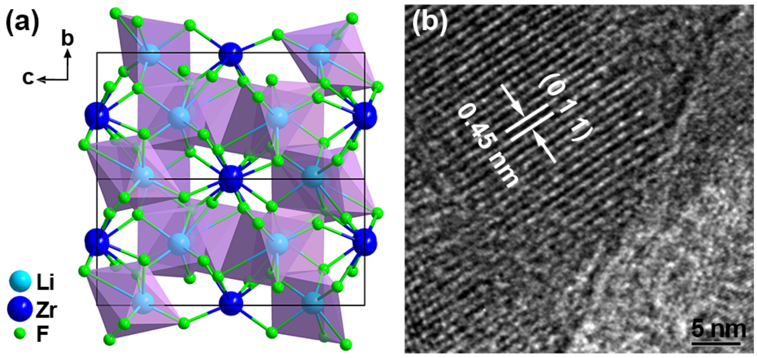

Figure S3. (a) Monoclinic-phase Li_2ZrF_6 that features a 3D close-packed crystal structure. (b) High-resolution TEM (HRTEM) image of the as-prepared solid Li_2ZrF_6:Yb/Er UCNCs, displaying clear lattice fringes with an observed d-spacing of 0.45 nm, in good agreement with the lattice spacing of the (011) plane of monoclinic-phase Li_2ZrF_6.

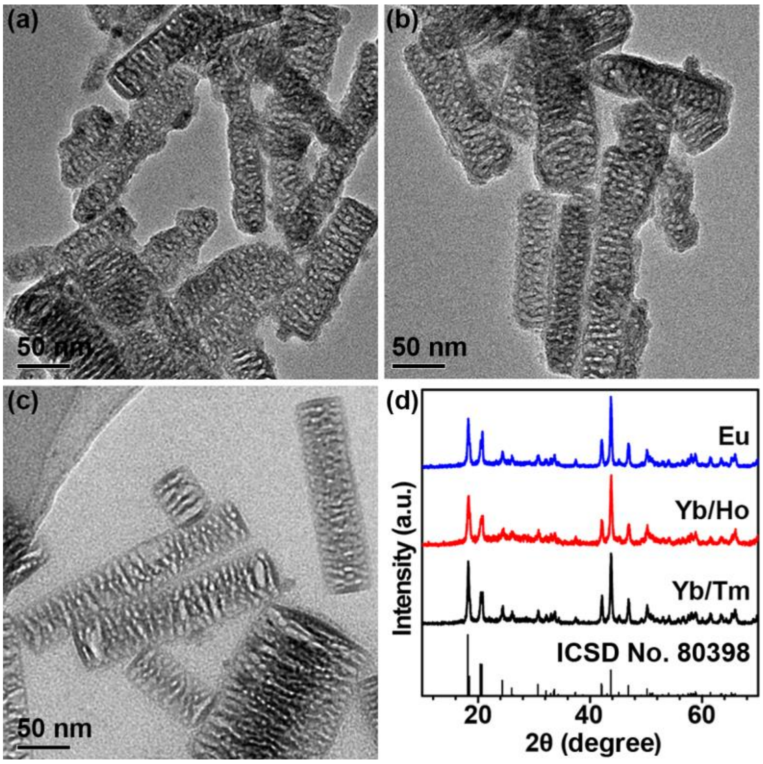

Figure S4. TEM images for the as-synthesized (a) Yb/Tm, (b) Yb/Ho and (c) Eu doped Li_4ZrF_8 NCs, showing their similar hollow peasecod-like morphologies as that of Li_4ZrF_8:Yb/Er UCNCs. (d) XRD patterns for the as-synthesized Yb/Tm, Yb/Ho and Eu doped Li_4ZrF_8 NCs. All the XRD peaks can be indexed in accordance with orthorhombic-phase Li_4ZrF_8 (ICSD No. 80398).

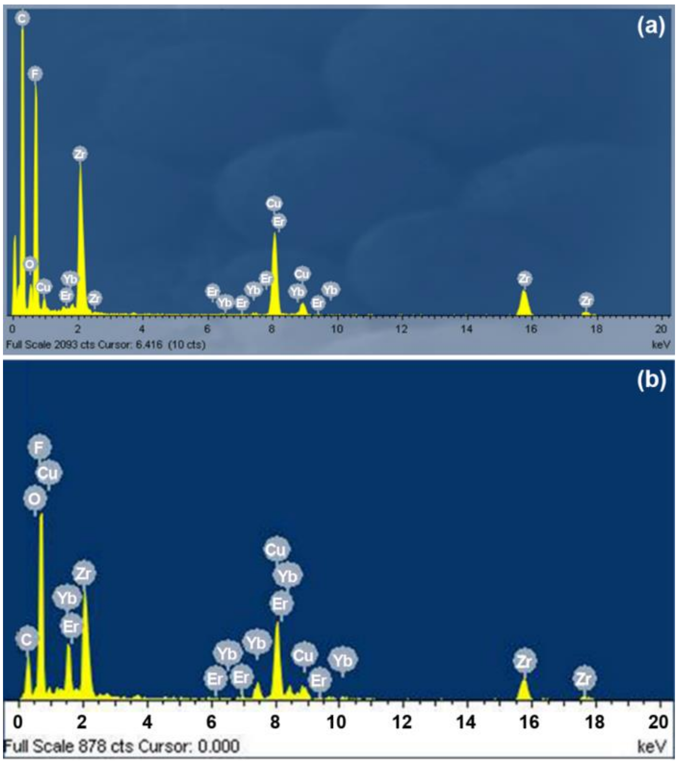

Figure S5. Energy-dispersive X-ray spectroscopy (EDS) analysis for the as-prepared (a) hollow Li_4ZrF_8:Yb/Er and (b) solid Li_2ZrF_6:Yb/Er UCNCs, revealing the presence of host elements of Zr, F and the dopants of Yb and Er in both UCNCs. The absence of host element of Li in the UCNCs is ascribed to its small atomic number (M=3) that cannot be detected by EDS.

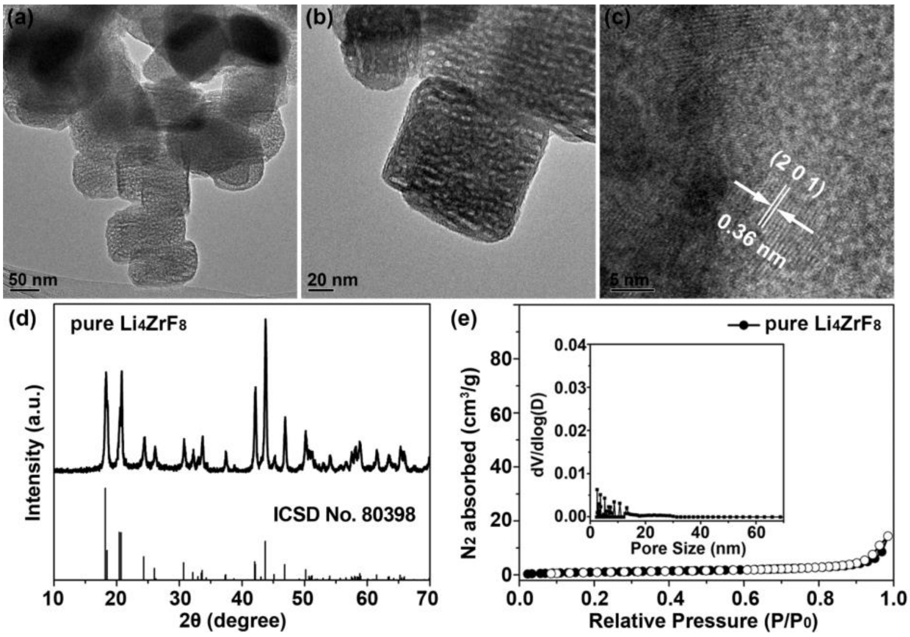

Figure S6. (a-b) TEM images of the as-prepared pure Li_4ZrF_8 NCs at different magnifications, and (c) their corresponding HRTEM image displaying the clear lattice fringes with an observed d-spacing of 0.36 nm, which is in good agreement with the lattice spacing of the (201) plane of orthorhombic-phase Li_4ZrF_8. (d) XRD pattern of the as-prepared pure Li_4ZrF_8 NCs, showing that all the diffraction peaks can be well indexed into orthorhombic-phase Li_4ZrF_8 (ICSD No. 80398). (e) N_2 adsorption-desorption isotherms and corresponding pore-size distribution (inset) for the pure Li_4ZrF_8 NCs at 77 K, indicating that no apparent pores can be detected in these NCs.

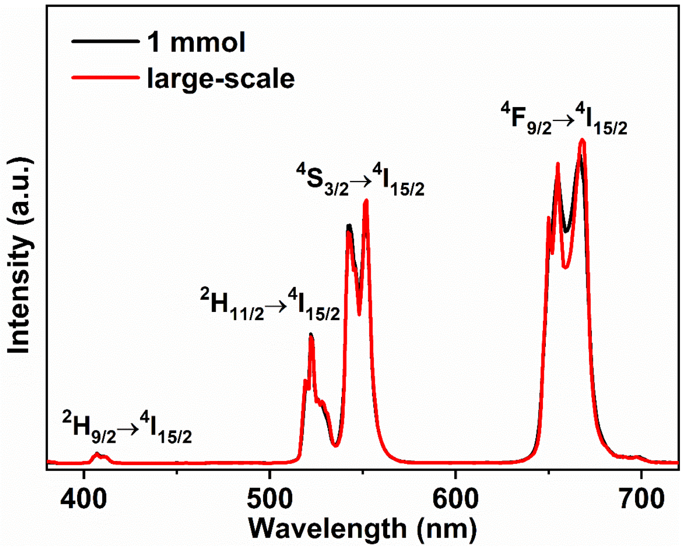

Figure S7. Comparison of UCL spectra for the as-prepared 1 mmol (black) and large-scale (red) peasecod-like hollow Li_4ZrF_8:Yb/Er UCNCs under excitation of a 980-nm diode laser, in which the luminescence intensities are found to be nearly identical, suggesting that the UCL performance for the hollow Li_4ZrF_8:Yb/Er UCNCs were preserved during scaling up.

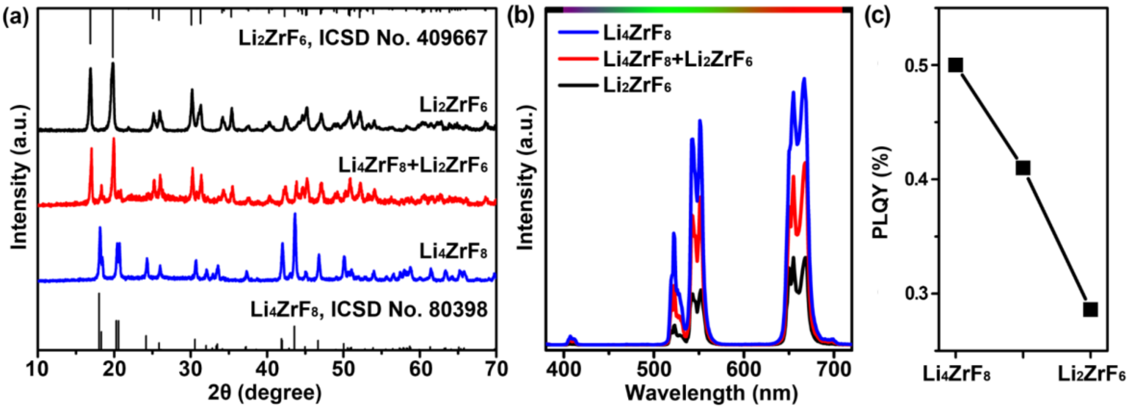

Figure S8. (a) XRD patterns for the mixed lithium zirconium fluoride UCNCs with different Li_4ZrF_8:Yb/Er and Li_2ZrF_6:Yb/Er contents and their corresponding (b) UCL patterns and (c) photoluminescence quantum yields (PLQYs). The phase transformation from hollow Li_4ZrF_8:Yb/Er to solid Li_2ZrF_6:Yb/Er UCNCs can be well evidenced by using the XRD analysis (Figure S8a). It was found that both the overall UCL intensity and PLQY for the hollow Li_4ZrF_8:Yb/Er UCNCs were larger than those of the solid Li_2ZrF_6:Yb/Er counterparts regardless of their much larger surface-to-volume (S/V) ratio, which clearly demonstrates that the surface quenching associated with the large S/V ratio has no significant impact on the UCL performance of the hollow Li_4ZrF_8:Yb/Er UCNCs we prepared.

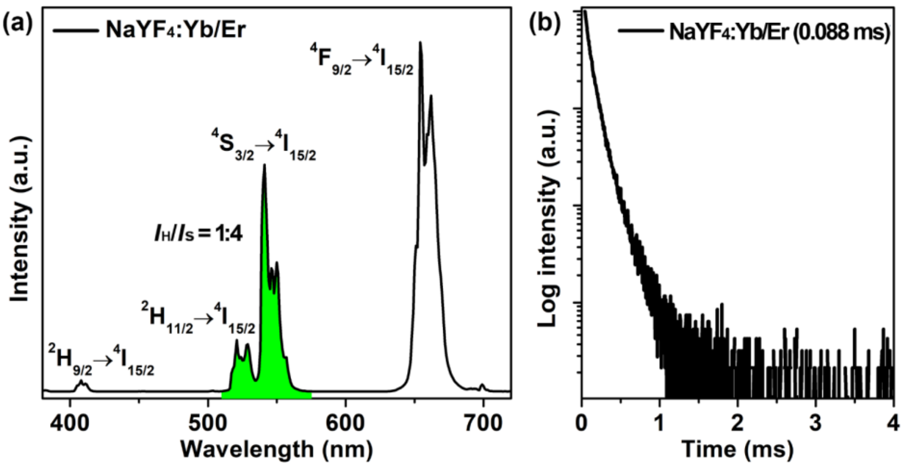

Figure S9. (a) Typical UCL spectrum and (b) corresponding $^2H_{11/2}$ lifetime of Er^{3+} of the as-prepared solid β-$NaYF_4$:Yb/Er UCNCs under excitation of a 980-nm diode laser. Characteristic UCL bands arising from the $^2H_{9/2}\rightarrow{}^4I_{15/2}$, $^2H_{11/2}$, $^4S_{3/2}\rightarrow{}^4I_{15/2}$, and $^4F_{9/2}\rightarrow{}^4I_{15/2}$ transitions of Er^{3+} for the solid β-$NaYF_4$:Yb/Er UCNCs were observed. The luminescence intensity ratio (LIR) of two green emissions (I_H/I_S) and $^2H_{11/2}$ lifetime of Er^{3+} for the solid β-$NaYF_4$:Yb/Er UCNCs are found to be virtually identical to those of the solid Li_2ZrF_6:Yb/Er UCNCs.

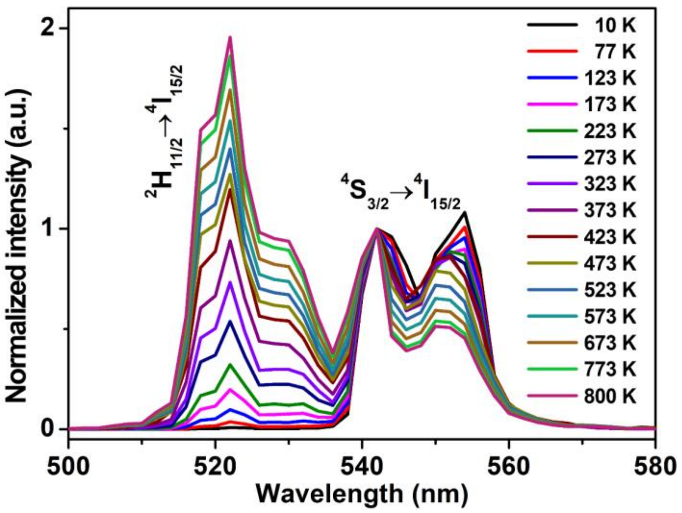

Figure S10. Temperature-dependent UCL spectra of the as-prepared solid Li_2ZrF_6:Yb/Er UCNCs recorded at 10-800 K, in which the peaks are normalized at 542 nm. The LIR of I_H/I_S of Er^{3+} for the solid Li_2ZrF_6:Yb/Er UCNCs increased with rising temperature, which is identical to the case of the hollow Li_4ZrF_8:Yb/Er UCNCs, as a result of enhanced thermal population of the $^2H_{11/2}$ state from the $^4S_{3/2}$ state at higher temperature.

Table S1. Calculated bond lengths for pure, Yb^{3+}, Er^{3+} and Yb^{3+}/Er^{3+} doped Li_4ZrF_8 crystals.

Bond distances (Å)	Li_4ZrF_8	Li_4ZrF_8:Er	Li_4ZrF_8:Yb	Li_4ZrF_8:Yb/Er
Li-F	1.9409	1.9375	1.9321	1.9238
	1.9735	1.9617	1.9632	1.9579
	1.9893	1.9981	2.0011	1.9903
	2.0890	2.0533	2.0521	2.0546
	2.2227	2.1396	2.1403	2.1827
	2.2229	2.1891	2.1700	2.2026
Zr-F	2.0586	2.0614	2.0604	2.0619
	2.0715	2.0760	2.0769	2.0805
	2.1307	2.1292	2.1273	2.1265
	2.1307	2.1386	2.1396	2.1440
	2.1439	2.1411	2.1425	2.1489
	2.1439	2.1531	2.1514	2.1627
	2.2833	2.2633	2.2615	2.2515
	2.2833	2.2818	2.2789	2.2716
Er-F		2.1668		2.1694
		2.1696		2.1752
		2.1784		2.1754
		2.1947		2.1783
		2.2203		2.2195
		2.2370		2.2212
		2.3081		2.2961
Yb-F			2.2062	2.2000
			2.2205	2.2149
			2.2217	2.2183
			2.2262	2.2285
			2.2517	2.2485
			2.2734	2.2678
			2.3534	2.3470

Table S2. Comparison of the calculated crystal lattice parameters for pure, Yb^{3+}, Er^{3+} and Yb^{3+}/Er^{3+} doped Li_4ZrF_8 crystals with the experimental data of their pure counterparts from ICSD database.

	Li_4ZrF_8[a]	Li_4ZrF_8	Li_4ZrF_8:Er	Li_4ZrF_8:Yb	Li_4ZrF_8:Yb/Er
Space group	Pnma	Pnma	Pnma	Pnma	Pnma
a/Å	9.738	9.677	9.689	9.715	9.726
b/Å	9.747	9.698	9.725	9.701	9.715
c/Å	11.516	11.465	11.539	11.545	11.616
α	90	90	89.8	89.8	89.6
β	90	90	90.1	90.1	90.4
γ	90	90	89.9	89.9	89.8
V/Å^3	1093.07	1075.94	1087.23	1088.06	1097.44

[a]The experimental crsytal lattice parameters for Li_4ZrF_8 from ICSD No. 80398. As shown in Table S2, the calculated crystal lattice parameters for pure and Ln^{3+}-doped Li_4ZrF_8 crystals are well consistent with the experimental data, which thereby demonstrates the reliability of our calculations.

Table S3. Calculated formation energies (defined as the energy difference per unit cell between the nanocrystal and isolated atoms) for pure, Yb^{3+}, Er^{3+} and Yb^{3+}/Er^{3+} doped Li_4ZrF_8 crystals by using the first-principle calculations.

Crystal	Content of dopant (mol%)	Formation energy per atom (eV)
Li_4ZrF_8	0	-3.3328
Li_4ZrF_8:Er	12.5	-3.3595
Li_4ZrF_8:Yb	12.5	-3.3155
Li_4ZrF_8:Yb/Er	12.5 (Yb/Er = 1)	-3.4034

The formation energies (E_{Form}) of the defect doped system in charge state q can be calculated according to the following equation.[1]

$$E_{Form}(q) = E_{tot}(defect, q) - E_{tot}(perfect) - \Sigma\eta_i\mu_i + q(E_F + E_V + \Delta V)$$

Where q is the number of electrons transferred in informing the defected supercell; $E_{Form}(q)$ is the defect formation energy of the system with charge q; $E_{tot}(defect,q)$ and $E_{tot}(perfect)$ are the total energies of the defected and perfect supercells, respectively; for component i, n_i indicates that the number of atoms of type i that have been added to ($n_i > 0$) or removed from ($n_i < 0$) the perfect supercell, μ_i is the corresponding chemical potential; E_F and E_V represent the Fermi energy and valence band maximum (VBM) for the perfect supercell, respectively. ΔV is a correction item of the electrostatic potentials between the supercells with and without dopants.

In our cases, the charge balance was taking into consideration for these Ln^{3+}-doped systems. Namely, the replacement of a zirconium ion by a rare earth ion was accompanied with the loss of one adjacent fluoride ion to form fluoride vacancy. The formation energies of these doped systems were simplified as following:

$$E_{Form} = E_{tot}(defect) - E_{tot}(perfect) - \Sigma\eta_i\mu_i$$

As presented in Table S3, the formation energy per atom of Li_4ZrF_8:Yb/Er decreased by about 0.07 eV in comparison with the pure Li_4ZrF_8 system, indicating that the hetero-valence

doping of Yb^{3+}/Er^{3+} into the crystal lattice of Li_4ZrF_8 matrix could effectively promote the energetical stability of Yb^{3+}/Er^{3+} doped system.

Table S4. Actual Yb^{3+} and Er^{3+} doping contents determined from the inductively coupled plasma atomic emission spectroscopy (ICP-AES) for hollow Li_4ZrF_8:Yb/Er and solid Li_2ZrF_6:Yb/Er UCNCs doped with 20 mol% Yb^{3+} and 2 mol% Er^{3+}.

Sample	Nominal Yb/Er doping content (mol%)	Actual Yb/Er doping content from ICP-AES (mol%)
Li_4ZrF_8:Yb/Er	20/2	19.8 ± 0.9/1.8 ± 0.2
Li_2ZrF_6:Yb/Er	20/2	18.7 ± 1.2/2.1 ± 0.3

References

[1] C. G. Van de Walle, J. Neugebauer, *J. Appl. Phys.*, **2004**, *95*, 3851.
